# Intravenous Serum Treatment of Epidemic Cerebrospinal Meningitis

**Published:** 1918

**Authors:** 


***Intravenous Serum Treatment of Epidemic Cerebrospinal Meningitis.*** By W. W. Herrick, M. R. C. Abs. from the *Archives of Internal Medicine.* April 15, 1918.

The author believes that the meningeal aspect of meningococcus infection has attracted general attention to the neglect of other important considerations, especially the point that meningococcus infections are primarily septic in nature and that meningitis is merely a secondary complication. The premeningitic stage of the sepsis especially has been neglected. In this stage, the author believes, lies the key to early diagnosis and treatment.

The author reports 208 cases in about half of which primary meningococcus sepsis was observed before selective action on the meninges was established. Intravenous serum therapy was thereby placed on a firm basis, with a notable reduction of mortality. Men with any suggestive symptoms were at once removed to a special hospital as suspects, and so the early stages of the disease could easily be studied.

The early symptoms are those of a generalized infection. This stage averages about 48 hours but may be much longer or shorter. The evidences of this stage are (1) clinical and (2) bacteriological.

*Clinical symptoms.* — Fever rarely rises above 102° F. (generally ranging from 99° to 101° F). The pulse is slow with vegal irregularities. There is a characteristic attitude, manner, and facies. The patient is not irritable. He resist, prefers to be left alone, lies on his side with thighs and knees drawn up, and shields his face with a covering. He is sensitive to cold and resents exposure. The patient avoids all effort even in speech. He has a vacant look of apathy and dulness.

The veins of the temporal region and forehead are full, the eyeballs are tender, and cyanosis of the face is most common, especially noticeable in the margins of the ears, often with a sharp line of demarcation. The pupils are almost always dilated. Some cases show retinal edema and obscurations of the disk margins and fulness of the retinal veins.

In many cases there is an upper respiratory tract infection, a coryza, pharyngitis, tonsillitis, laryngitis; rarely, bronchitis. In four cases a pneumonia was observed. This respiratory involvement is not a mere coincidence, but demands serious study. Oral secretions are viscid, the tongue coated, and the breath heavy.

Skin manifestations are most important. Very early there may be a more or less diffuse mottling, giving a lead-colored appearance. *Tache cérébrale* is constant but of slight diagnostic value. Macular rash resembling early chicken pox or rose spots has been observed in a few cases, generally of the less severe type. The predominant skin sign was the petechial rash about the shoulder or the pelvic girdle, and less frequently over the trunk, extremities.

